# Functional asymmetry of the transmembrane subunits YbhS and YbhR in the tetracycline-inducible ABC transporter YbhFSR

**DOI:** 10.3389/fmicb.2026.1959624

**Published:** 2026-10-23

**Authors:** Zhenyue Feng, Chaohuan Yang, Hao Wen, Meng Wu, Shengguang Feng, Wei Zheng, Defu Liu

**Affiliations:** 1 College of Agronomy and Life Sciences, Zhaotong University, Zhaotong, China; 2 Yunnan Engineering Research Center of Green Planting and Processing of Gastrodia, Zhaotong University, Zhaotong, China; 3 Institute for Advanced Study on Ecological Agriculture and Biodiversity of the Yunnan-Guizhou Plateau, Zhaotong University, Zhaotong, China; 4 Yunnan Key Laboratory of Smart Villages and Agri-Cultural-Tourism Integration, Zhaotong University, Zhaotong, China; 5 College of Agronomy and Biotechnology, Yunnan Agricultural University, Kunming, China

**Keywords:** ABC transporter, *Escherichia coli*, functional asymmetry, tetracycline resistance, YbhFSR efflux pump

## Abstract

**Objective:**

The *Escherichia coli* ABC (ATP-binding cassette) transporter YbhFSR has an unusual subunit architecture: its two nucleotide-binding domains (NBDs) are fused within a single polypeptide YbhF, while the transmembrane domains (TMDs) are formed by two separate subunits YbhS and YbhR. This study sought to investigate the functional differences between YbhS and YbhR during tetracycline (TC) efflux, and to characterize the transcriptional regulation of the ybhFSR operon under TC stress.

**Methods:**

A series of *E. coli* knockout strains (ΔybhF, ΔybhS, ΔybhR, ΔybhSR, ΔybhFSR) were generated via λ-Red homologous recombination. qRT-PCR was used to profile transcriptional responses of ybhF, ybhS, and ybhR under a gradient of TC concentrations in wild-type and ΔacrB backgrounds. Susceptibility to four tetracyclines (TC, OTC, CTC, DOX) was determined by broth microdilution and disk diffusion. Cross-species sequence alignments across seven Enterobacteriaceae genera compared evolutionary conservation between YbhS and YbhR.

**Results:**

Tetracycline triggered dose-dependent transcriptional upregulation of ybhF, ybhS and ybhR, whereby ybhR was induced to a greater extent than ybhS across all tested concentrations. The transcriptional gap between ybhR and ybhS was markedly enlarged in the ΔacrB strain, showing a significant divergence in inducibility (23.8-fold for ybhR vs. 10.2-fold for ybhS at 8 μg/mL TC). Deletion of ybhR led to markedly increased susceptibility to all four tetracyclines (TC, OTC, CTC, DOX), while *E. coli* ΔybhS cells exhibited MIC (minimum inhibitory concentration) values indistinguishable from the wild type. The ΔacrB ΔybhF double mutant yielded a TC MIC of 0.125 μg/mL, considerably lower than the ΔacrB single mutant (0.25 μg/mL) and wild-type strain (1 μg/mL). Cross-species alignments across seven Enterobacteriaceae genera showed that YbhR had higher average sequence identity than YbhS (96.20% versus 93.89%); however, this difference did not reach statistical significance (Wilcoxon signed-rank test, *p* = 0.0625; paired t-test, *p* = 0.75).

**Conclusion:**

Transcription of the ybhFSR operon is induced by TC in a dose-dependent fashion, and this induction is enhanced in the absence of AcrAB. YbhS and YbhR exhibit functional asymmetry at both transcriptional and physiological levels: YbhR acts as the core TMD subunit supporting TC export, whereas YbhS plays a dispensable role in tetracycline efflux under the tested conditions. The consistently higher sequence conservation of YbhR relative to YbhS is consistent with its dominant physiological role, although this trend did not reach statistical significance. Notably, this demonstrates TMD functional asymmetry in a Gram-negative transporter with fused NBDs, indicating that TMD specialization can occur independently of NBD architecture even when NBD asymmetry is hard-wired into the fused YbhF polypeptide.

## 1 Introduction

Bacterial antimicrobial resistance (AMR) is one of the most serious challenges facing global public health in the 21st century. The Global Burden of Disease study predicts that without effective intervention strategies, antimicrobial resistance could result in approximately 10 million annual deaths by 2050 (Nazarov, 2022; Kariuki, 2024). Enhanced expression of efflux pumps represents one of the primary mechanisms underlying antimicrobial resistance in Gram-negative bacteria (Athar et al., 2023; Novelli and Bolla, 2024; Weston et al., 2018). These pumps extrude diverse compounds and contribute to stress tolerance, virulence, and biofilm formation (Gaurav et al., 2023; Almeida da Silva et al., 2025; Nanjan and Bose, 2024; Sharma et al., 2023).

The ABC (ATP-binding cassette) superfamily comprises the only class of primary active efflux pumps powered by ATP hydrolysis. Their core architecture consists of two NBDs and two TMDs. TMDs and NBDs establish molecular communication through coupling helices at their mutual interface. However, whether the two TMD subunits contribute equally to substrate transport, or adopt distinct roles, remains unclear. These helices mediate allosteric coupling. Energy released from ATP hydrolysis is transmitted to the substrate-binding pocket, and coordinated transmembrane transport of substrates is achieved (Higgins, 1992; Davidson et al., 2008; Rees et al., 2009; George and Jones, 2012; Zolnerciks et al., 2011). Bacterial ABC transporters widely mediate the transmembrane translocation of drugs, lipids, peptides and metabolites. They also serve as important determinants of bacterial multidrug resistance and virulence (Akhtar and Turner, 2022; How et al., 2025). Despite their well-recognized importance, substrate specificity and mechanisms governing subunit division of ABC transporters remain to be elucidated.

The *ybhFSR* operon in *E. coli* K-12 encodes a typical ABC-type efflux pump. *ybhF* encodes two NBD domains within a single polypeptide, while *ybhS* and *ybhR* each encode one TMD subunit. Our previous work showed the substrate spectrum of YbhFSR. We confirmed that this transporter expels tetracycline-class antibiotics including TC, OTC, CTC, DOX, as well as ethidium bromide and Hoechst 33342. We also identified its Na^+^(Li^+^)/H^+^ antiport activity. This finding establishes YbhFSR as a bifunctional transporter (Feng et al., 2020). Two questions remain: how TC regulates *ybhFSR* transcription, and whether YbhS and YbhR have distinct roles. In *E. coli*, the RND-family AcrAB-TolC system is the major multidrug efflux pump and the principal determinant of intrinsic resistance to tetracyclines (Li et al., 2015; Weston et al., 2018), and its loss sensitizes cells to secondary efflux systems such as YbhFSR.

The second question merits particular investigation. Functional asymmetry between TMD subunits has been established as a prevalent design principle in heterodimeric ABC efflux pumps. *Bacillus subtilis* BmrCD represents the best structurally resolved heterodimeric ABC transporter. Cryo-electron microscopy data reveal clear functional divergence between its two TMD subunits BmrC and BmrD, with respect to substrate-binding cavity configuration and the efficiency of ATP hydrolysis-dependent allosteric coupling. BmrC dominates the formation of the core substrate-binding pocket and undergoes most transmembrane helix conformational rearrangements, whereas BmrD mainly supports the maintenance of protein scaffold and forms only weak interactions with substrates (Tang et al., 2023; Tang et al., 2025). DEER (Double Electron–Electron Resonance) conformational dynamics analyses of the thermophilic bacterial transporter TM287/288 show that its two NBDs contain one degenerate, inactivated ATP-binding site. In the absence of nucleotides, asymmetric dissociation of NBDs occurs. Conformational changes of NBDs cannot be synchronously transmitted to the two TMDs. Transmembrane subunits thus differ in their efficiency to receive allosteric signals. That study also conducted parallel comparisons with BmrCD and proposed that although both belong to heterodimeric ABC transporters with degenerate NBDs, their kinetic features of ATP-driven conformational transitions differ across species (Timachi et al., 2017). These findings collectively suggest that differential contribution of TMD subunits represents an evolutionarily conserved design rule for heterodimeric ABC multidrug efflux pumps. However, whether this asymmetry principle extends to Gram-negative transporters with fused NBDs remains untested. Current experimental evidence for TMD functional asymmetry is mostly obtained from Gram-positive ABC transporters.

Gram-negative ABC exporters exhibit a distinctive domain organization: two NBD modules are tandem fused within a single polypeptide chain, as exemplified by *E. coli* YbhF, while their TMD moieties assemble from two independent protein subunits. To date, no *in vivo* assays have validated unequal functional contributions of paired TMD subunits within transporters bearing this distinct domain layout.

YbhFSR differs structurally from the Gram-positive prototypes BmrCD and TM287/288. The functional asymmetry observed in this transporter appears to arise primarily from its two TMD paralogs YbhS and YbhR. By contrast, the single YbhF polypeptide harbors two sequential NBD domains whose arrangement recapitulates the dual-NBD architecture seen in canonical homodimeric ABC efflux pumps.

Sequence analysis confirmed that these two NBDs are not functionally equivalent. NBD1 retains the canonical conserved motifs of classical ABC transporters, whereas the LSGGQ motif of NBD2 exhibits obvious substitutions (LSGGM → LRGRA, S → R and G → R), implying that NBD2 is potentially catalytically degenerate. This motif comparison was reported in our previous study (Feng et al., 2026).

We hypothesized that tetracycline stress drives concentration-dependent activation of the *ybhFSR* operon with distinct induction strengths for *ybhS* and *ybhR*, and that the two TMD subunits carry unequal roles in tetracycline export. Specifically, we predicted YbhR would function as the primary subunit for TC extrusion, whereas YbhS would only support transporter assembly and structural stability.

We tested this hypothesis by constructing a series of gene deletion strains using λ-Red homologous recombination (Datsenko and Wanner, 2000). Wild-type and Δ*acrB* strains were exposed to a gradient of TC concentrations. Quantitative real-time PCR (qRT-PCR) was applied to detect transcriptional responses of *ybhF*, *ybhS* and *ybhR*. Broth microdilution and Clinical and Laboratory Standards Institute (CLSI)-standard disk diffusion assays were performed to systematically compare antibiotic susceptibility of all mutant strains toward four tetracyclines. Cross-species sequence alignment was conducted to evaluate evolutionary conservation of YbhS and YbhR. Transcriptional responses, functional phenotypes and evolutionary conservation data were integrated in our analysis. This study aims to clarify the induction pattern of YbhFSR during TC resistance and uncover functional asymmetry between YbhS and YbhR, while supplying new experimental evidence for the evolutionary biology of subunit functional differentiation in ABC transporters.

## 2 Materials and methods

### 2.1 Bacterial strains and culture conditions

All strains are derivatives of *E. coli* K-12. We constructed single-gene deletion strains *E. coli* Δ*ybhF*, *E. coli* Δ*ybhS*, *E. coli* Δ*ybhR*, the double-gene deletion strain *E. coli* Δ*ybhSR*, and the triple-gene deletion strain *E. coli* Δ*ybhFSR*, using wild-type *E. coli* K-12 as the genetic background and the λ-Red homologous recombination system (Datsenko and Wanner, 2000). The drug-susceptible strain *E. coli* Δ*acrB* and double-gene deletion strain *E. coli* Δ*acrB*Δ*ybhF* were generated previously by our research group (Feng et al., 2020). Strains and plasmids used in this study are listed in Table 1. All strains were cultured in Luria-Bertani (LB) liquid medium at 37 °C with shaking at 180 rpm. Chloramphenicol was added at a final concentration of 25 μg/mL for antibiotic selection during λ-Red homologous recombination.

**Table 1:** Bacterial strains and plasmids used in this study.

Bacterial strains and plasmids	Genotype/description	Reference or source
Bacterial strains		
*E. coli* K‑12 MG1655	Wild type	Lab collection
*E. coli* DH5α	F– φ80lacZΔM15 Δ(lacZYA-argF)U169 recA1 endA1 hsdR17	TaKaRa
*E. coli* Δ*ybhS*	*ybhS* deletion mutant	This study
*E. coli* Δ*ybhR*	*ybhR* deletion mutant	This study
*E. coli* Δ*ybhSR*	*ybhSR* deletion mutant	This study
*E. coli* Δ*ybhFSR*	*ybhFSR* deletion mutant	This study
*E. coli* Δ*ybhF*	*ybhF* deletion mutant	Feng et al. (2020)
*E. coli* Δ*acrB*	*acrB* deletion mutant	Feng et al. (2020)
*E. coli* Δ*acrB*Δ*ybhF*	*acrB and ybhF* deletion mutant	Feng et al. (2020)
*E. coli*/pET28a-*ybhF*	pET 28a-*ybhF*, Kan^r^	Feng et al. (2020)
Plasmids		
pKD46	bla (Ap^r^), a helper plasmid for expression of Redλ recombinase	Lab collection
pKD3	Template plasmid for a chloramphenicol resistance gene	Lab collection
PCP20	bla (Ap ^r^) cat (Cm ^r^), a plasmid for expression of FLP recombinase	Lab collection

### 2.2 Antibiotics and reagents

#### 2.2.1 Antimicrobial agents and disk diffusion materials

The antimicrobial agents used in this study included TC, OTC, CTC, DOX, ethidium bromide (EB), Hoechst 33342, cefoperazone, cefazolin, streptomycin, ampicillin, roxithromycin, chloramphenicol, rifampicin, norfloxacin, sodium deoxycholate, sodium cholate, ofloxacin, adriamycin, daunorubicin, acriflavine, and quinine. All compounds were purchased from Beijing Coolaber Technology Co., Ltd. (Coolaber, Beijing). Drug susceptibility disks (TC 30 μg/disk, OTC 30 μg/disk, CTC 30 μg/disk, DOX 30 μg/disk) were obtained from Hangzhou Binhe Microbial Reagent Co., Ltd.

Antimicrobial stock solutions were prepared as follows. Each agent was dissolved in sterile deionized water or an appropriate solvent (dimethyl sulfoxide was used for compounds with low water solubility) to make stock solutions. The stock solutions were filter-sterilized through 0.22 μm membranes, aliquoted, and stored at −20 °C in the dark. Working solutions were prepared by twofold serial dilution in MH (Mueller-Hinton) broth immediately before use.

#### 2.2.2 Media and selective antibiotics

Luria-Bertani (LB) liquid medium (tryptone 10 g/L, yeast extract 5 g/L, NaCl 10 g/L) and MH liquid and agar media were prepared according to standard formulas and sterilized by autoclaving. Cation-adjusted Mueller–Hinton broth (CAMHB; QDRS BIOTEC, Qingdao, China; catalog no. 11865) and Mueller–Hinton agar (MHA; QDRS BIOTEC, Qingdao, China; catalog no. 10706) were prepared according to the manufacturer’s instructions and sterilized by autoclaving at 121 °C for 10 min. Antibiotics were used at the following working concentrations for selection: chloramphenicol at 25 μg/mL (for λ-Red homologous recombination selection), kanamycin at 50 μg/mL (for pET28a-*ybhF* plasmid selection), and ampicillin at 100 μg/mL (for pKD46 and pCP20 plasmid selection).

#### 2.2.3 RNA extraction and quantitative real-time PCR reagents

Total RNA was extracted using the Axygen^®^ Bacterial RNA Extraction Kit (Axygen Biosciences, USA) according to the manufacturer’s instructions. Reverse transcription was performed with the Toyobo ReverTra Ace^®^ qPCR RT Master Mix (with gDNA Remover, Toyobo, Japan) to remove genomic DNA and synthesize cDNA. Quantitative real-time PCR was carried out using the LightCycler^®^ 480 SYBR Green I Master Mix (Roche, Switzerland) for SYBR Green dye-based detection. RNase-free water was purchased from TaKaRa (Dalian, China). The specific primers used for qRT-PCR (Q-*ybhF*, Q-*ybhS*, Q-*ybhR*, Q-*acrB*, Q-*gapA*) are listed in Supplementary Table S1 and were synthesized by Sangon Biotech (Shanghai) Co., Ltd.

### 2.3 Gene cloning and construction of deletion mutant strains

Gene sequences of *ybhR* (Gene ID: 945403) and *ybhS* (Gene ID: 945411) were retrieved from NCBI GenBank using the *E. coli* K-12 MG1655 reference genome. Specific primers were designed with Primer 5.0 and are listed in Supplementary Table S1. The full-length coding sequences of *ybhS* and *ybhR* were amplified by PCR from *E. coli* K-12 genomic DNA, and the amplicons were sequenced to confirm their accuracy.

To determine the role of each subunit in TC efflux, we constructed a series of gene deletion mutants using the λ-Red recombination system (Datsenko and Wanner, 2000). We first transformed pKD46 into *E. coli* K-12 to prepare competent cells expressing Red recombinase. Using pKD3 as the template, we PCR-amplified chloramphenicol/kanamycin resistance cassettes flanked by FRT sites, with primers carrying 45–50 bp homology arms matching the target gene flanks. The purified cassettes were electroporated into *E. coli* K-12 cells carrying pKD46. Red recombinase mediated replacement of the target gene with the resistance cassette. Transformants were selected on antibiotic-containing plates and screened by colony PCR with diagnostic primer pairs. Positive clones were further confirmed by sequencing. Confirmed mutants were cured of pKD46 by culturing at 42 °C, then transformed with pCP20. FLP recombinase was induced at 42 °C to remove the resistance marker between the two FRT sites.

We used the pET28a-*ybhF* overexpression strain previously constructed in our laboratory (Feng et al., 2020) to assess YbhF function in TC efflux. The full-length *ybhF* coding sequence from *E. coli* K-12 was cloned into pET28a(+) (Novagen) between the *Bam* HI and *Hind* III sites, generating pET28a-*ybhF*. After sequencing confirmation, the plasmid was transformed into *E. coli* K-12 competent cells. Transformants were selected with kanamycin (50 μg/mL) and verified by colony PCR and sequencing. This strain was used as a complementation control in drug susceptibility assays.

### 2.4 Measurement of growth curves

Single colonies of wild-type and mutant strains (*E. coli* Δ*ybhS*, *E. coli* Δ*ybhR*, *E. coli* Δ*ybhSR*, *E. coli* Δ*ybhFSR*) were inoculated into 3 mL LB broth and incubated overnight at 37 °C with shaking at 180 rpm. Overnight cultures were diluted 1:100 in fresh antibiotic-free LB medium and further incubated at 37 °C with shaking at 180 rpm. The OD₆₀₀ was measured at 1-h intervals for 12 h using a spectrophotometer. At each sampling time, 1 mL aliquots were withdrawn from the growing culture. For each independent experiment performed on separate days, three parallel cultures were included as technical replicates. The entire assay was independently repeated three times (*n* = 3 biological replicates). Data are presented as mean ± SD.

### 2.5 Construction of TC gradually induced resistant strains

The transcriptional response of the *ybhFSR* operon under TC stress was characterized by gradually inducing wild-type *E. coli* K-12 and the drug-susceptible strain Δ*acrB* with TC. Single colonies were picked from MH agar plates, inoculated into 2 mL antibiotic-free MH broth, and incubated overnight at 37 °C with shaking at 180 rpm. The next day, cultures were diluted 1:100 into fresh LB medium containing TC at a starting concentration equal to the MIC of each strain (1 μg/mL for WT and 0.25 μg/mL for Δ*acrB*; Feng et al., 2020). After 12 h of incubation at 37 °C, the bacterial suspension was transferred at the same ratio into fresh LB medium with stepwise increased TC concentrations. For WT, the concentration was raised to 5, 10, 15, 20, and 25 μg/mL; for Δ*acrB*, it was raised to 2, 4, 6, and 8 μg/mL. At each step, the culture was passaged until growth was restored before the next concentration increase. Bacterial samples were collected at five concentrations along each gradient (5–25 μg/mL for WT and 2–8 μg/mL for Δ*acrB*) for RNA extraction and MIC measurement. Serial passaging continued until the MIC reached a plateau. Bacterial samples at each induction concentration were collected for RNA extraction and MIC measurement. Each concentration gradient was tested with three independent biological replicates and three technical replicates per test.

### 2.6 Total RNA extraction and quantitative real-time PCR (qRT-PCR)

Total RNA was extracted from wild-type *E. coli* K-12 and Δ*acrB* cultures at mid-log phase under each TC concentration using the Axygen Bacterial RNA Extraction Kit. About 0.5–2 × 10^9^ cells were collected by centrifugation at 6000 × g for 10 min. The supernatant was discarded, and the pellet was resuspended in 50 μL PBS, then transferred to a pre-cooled grinding tube and ground to powder. Buffer R-I (400 μL) was added, and the mixture was pipetted vigorously 8–10 times before transfer to a 1.5 mL tube. Buffer R-II (150 μL) was added, vortexed for 15–30 s, and centrifuged at 12000 × g, 4 °C for 5 min. The supernatant was mixed with 250 μL isopropanol, loaded onto the preparation column, and centrifuged at 6000 × *g*, 4 °C for 1 min. The column was washed with 500 μL Buffer W1A and then 700 μL Buffer W2. RNA was eluted with 70–100 μL RNase-free water. RNA concentration and purity (OD₂₆₀/₂₈₀ ratio, 1.9–2.1) were determined on a NanoDrop 2000 using 1 μL RNA. Genomic DNA removal and cDNA synthesis were performed with the Toyobo ReverTra Ace qPCR RT Master Mix (with gDNA Remover) according to the manufacturer’s instructions.

qRT-PCR was performed using SYBR Green I dye with cDNA as template. The 20 μL reaction contained 10 μL LightCycler 480 SYBR Green I Master Mix, 0.5 μL each of forward and reverse primers (10 μmol/L), 1 μL cDNA, and RNase-free water to volume. Primer sequences are listed in Supplementary Table S1. *gapA* served as the reference gene, and target genes included *ybhF*, *ybhS*, *ybhR* and *acrB*. The cycling conditions were: 95 °C for 10 min; 40 cycles of 95 °C for 10 s and 57 °C for 20 s; melting curve from 65 °C to 97 °C at 0.5 °C/s. Standard curves were generated from five 10-fold serial dilutions of cDNA to verify primer efficiency. Each sample was run in three technical replicates from three independent biological replicates. Relative expression was calculated by the 2^−ΔΔCt^ method (Livak and Schmittgen, 2001).

### 2.7 Disk diffusion susceptibility test

Disk diffusion assays followed CLSI standards. Overnight cultures were adjusted to 0.5 McFarland turbidity. A sterile cotton swab was dipped into the bacterial suspension and spread evenly onto MH agar plates. TC, OTC, CTC, and DOX disks were placed on the agar, spaced at least 24 mm apart. plates were incubated upside down at 35 °C for 18–24 h within 15 min of disk placement. Inhibition zone diameters (including the disk) were measured with calipers in millimeters. Each strain was tested in three independent biological replicates. Each plate in every biological replicate contained three antibiotic-susceptibility disks corresponding to TC, OTC, CTC and DOX treatments, respectively. Within each biological replicate, the measurement values obtained from the four disks on a single plate were not treated as technical replicates. The mean ± SD of the three independent biological replicates was used for statistical analysis.

### 2.8 Determination of minimum inhibitory concentration (MIC)

MIC of each strain against various antimicrobial agents was determined using the broth microdilution method. Stock solutions were prepared per CLSI guidelines. Serially diluted drugs (100 μL) were added to columns 1–11 of sterile 96-well plates. Column 12 served as controls: upper four wells received 200 μL MH broth without bacteria (negative control); lower four wells received 200 μL MH bacterial suspension without antibiotics (positive growth control). Single colonies were grown overnight in MH broth, adjusted to 0.5 McFarland standard (~10^8^ CFU/mL), diluted 1,000-fold in MH broth, and 100 μL was added to each well to a final concentration of approximately 10^5^ CFU/mL. Plates were incubated at 37 °C for 18–22 h, and OD₆₀₀ was read on a microplate reader. MIC was defined as the lowest drug concentration that completely inhibited visible growth. Each strain had three replicate wells, and the experiment was repeated three times.

### 2.9 Fluorometric assay for TC efflux activity

The TC efflux activity was determined via a fluorescence-based assay adapted from Feng et al. (2020), with slight adjustments. Briefly, wild-type *E. coli* K-12 and mutant strains (*E. coli* Δ*ybhS*, *E. coli* Δ*ybhR*, *E. coli* Δ*ybhSR*, *E. coli* Δ*ybhFSR*) were grown in LB medium to mid-log phase (OD₆₀₀ ≈ 0.8). Cells were collected by centrifugation, washed twice with Mg^2+^ buffer (pH 8.0; 10 mM Tris–HCl, 0.1 mM MgCl₂, 0.2% glucose), and resuspended in the same buffer to OD₆₀₀ of 0.8. TC was supplemented to a final concentration of 100 μg/mL to trigger efflux. This concentration was chosen to maintain consistency with our previous characterization of YbhFSR-mediated TC efflux (Feng et al., 2020) and to generate robust and reproducible fluorescence signals for efflux quantification. Extracellular fluorescence from exported TC was measured on a Tecan Infinite M200 PRO spectrofluorometer; excitation was set at 400 nm (9 nm slit width) and emission at 520 nm (20 nm slit width) (de Cristobal et al., 2006). Fluorescence intensity was acquired every 1 min over a 30-min measurement period. Wild-type *E. coli* K-12 was used as the positive control, and Δ*ybhFSR* served as the negative control. Each strain was tested in technical triplicate, and experiments were reproduced in three independent biological replicates.

### 2.10 Bioinformatics analysis

Amino acid sequences of YbhS and YbhR were downloaded from NCBI GenBank. We selected type strains from seven Enterobacteriaceae genera (*E. coli*, *Shigella*, *Enterobacter*, *Salmonella*, *Klebsie*lla, *Citrobacter*, and *Serratia*). Hereafter, genus names are abbreviated to their single-letter initials. Sequences of *E. coli* K-12 MG1655 served as references for local BLAST searches. We calculated protein sequence identities within each genus and obtained the identity difference between YbhR and YbhS. EMBOSS (European Molecular Biology Open Software Suite) Needle was used for global alignment of full-length YbhS and YbhR sequences to obtain identity and similarity scores. The EMBOSS Pepstats program calculated the proportion of aromatic residues (Phe, Tyr, Trp) and the Dayhoff statistic. We applied chi-square tests to evaluate intergroup differences in aromatic amino acid content. TMHMM 2.0 (TransMembrane Hidden Markov Model) predicted transmembrane topology. We split sequences into transmembrane helices and cytoplasmic/extracellular soluble segments, quantified the number and proportion of aromatic residues in each region, and compared aromatic residue composition and distribution patterns between YbhS and YbhR.

The sequence comparison of the YbhF NBD1 and NBD2 motifs (cited in Introduction and Discussion) was reported in our previous study (Feng et al., 2026) and is presented here as background rather than as a result of the present work.

### 2.11 Statistical analysis

All experiments were performed with at least three independent biological replicates, and data are presented as mean ± SD. GraphPad Prism was used for statistical analyses, and Origin (version 2023) for figure generation. Expression levels of *ybhS* and *ybhR* under identical TC concentrations were compared using unpaired *t*-tests within each concentration. Multigroup comparisons were analyzed by one-way ANOVA with Tukey’s *post hoc* test (for pairwise comparisons) or Dunnett’s *post hoc* test (for comparisons against the 0 μg/mL control). Inhibition zone data were evaluated by two-way ANOVA, whereas MIC values were determined directly without statistical testing. For growth curve analysis, data from three independent biological replicates (*n* = 3) were analyzed by repeated-measures two-way ANOVA with strain as the between-subject factor and time as the within-subject factor, followed by Tukey’s *post hoc* test for multiple comparisons. Sphericity was assessed and Greenhouse–Geisser correction was applied when required. Differences were considered significant at *p* < 0.05, highly significant at *p* < 0.01, and extremely significant at *p* < 0.001. Each biological replicate was performed with three technical replicates, and statistical analyses were based on the three independent biological replicates (*n* = 3). Given the limited sample size, non-significant differences should not be interpreted as evidence of equivalence.

## 3 Results

### 3.1 Validation of gene deletion strains

Full-length coding sequences of *ybhS* (1,134 bp) and *ybhR* (1,107 bp) were amplified by PCR and sequenced 100% identity with the NCBI reference sequence (Supplementary Figure S1, full-length PCR verification of *ybhS*/*ybhR*). Targeting cassettes for deletion of *ybhS*, *ybhR*, *ybhSR*, and *ybhFSR* were amplified from pKD3, yielding 1,041-bp products in each case. Agarose gel electrophoresis revealed single bands of the expected size for all constructs (Figures 1A–D, PCR products of knockout homologous arms).

**Figure 1:**
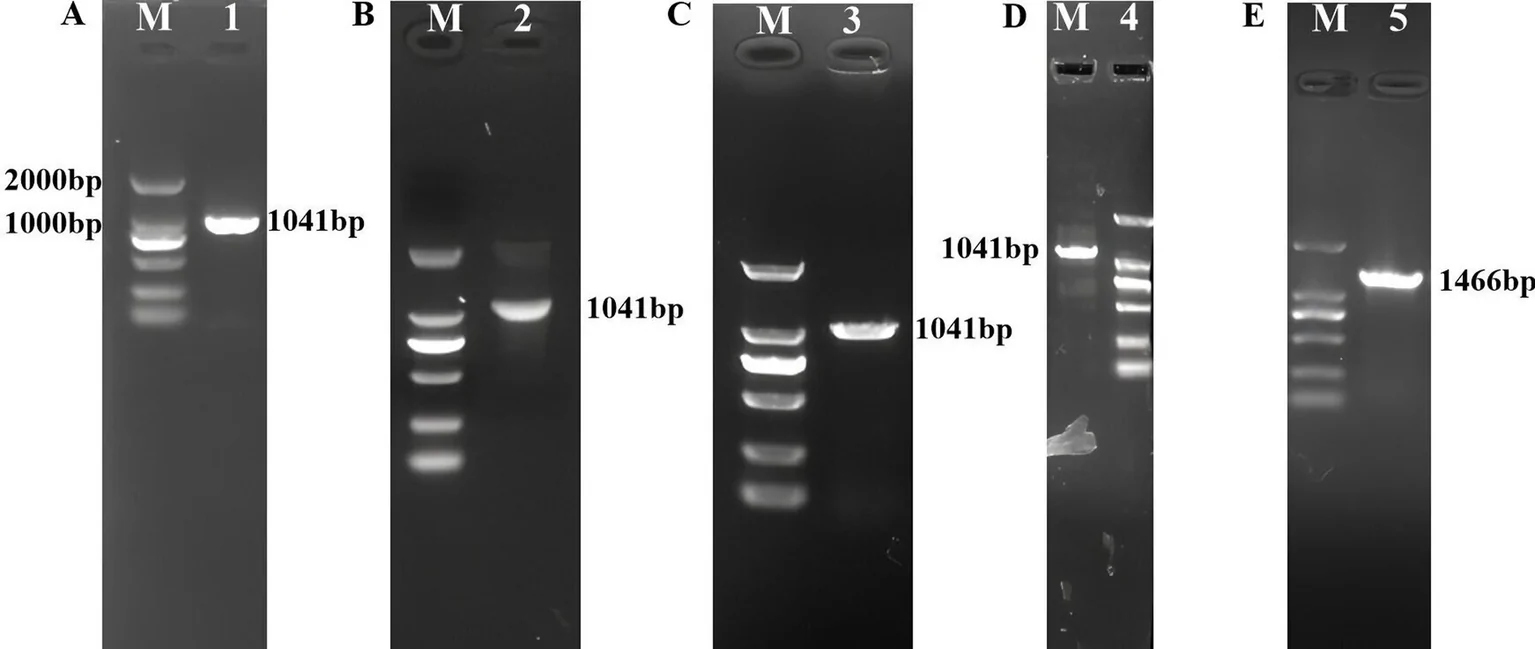
Analysis of λ-Red recombineering targeting cassettes and the pKD46 recombinase plasmid by 1% agarose gel electrophoresis. Representative gel images showing the PCR amplification products. **(A)** Lane 1: Targeting cassette for the Δ*ybhS* deletion. **(B)** Lane 2: Targeting cassette for the Δ*ybhR* deletion. **(C)** Lane 3: Targeting cassette for the Δ*ybhSR* double deletion. **(D)** Lane 4: Targeting cassette for the Δ*ybhFSR* triple deletion. **(E)** Lane 5: PCR verification of the pKD46 recombinase plasmid. Lane M: DL2000 DNA marker (from top to bottom: 2000, 1,000, 750, 500, 250, and 100 bp). Lanes 1–4 show the FRT-flanked resistance cassettes (expected size ~1,041 bp). Lane 5 shows the pKD46-specific amplicon (expected size ~1,466 bp). All experiments were repeated three times with similar results (n = 3 independent replicates). As this figure presents qualitative gel electrophoresis data, statistical tests and error bars are not applicable.

Plasmid pKD46 was introduced into *E. coli* K-12, and transformants were verified by PCR using pKD46-specific primers, which produced a 1,466 bp product (Figure 1E, pKD46 plasmid identification results). Competent cells harboring pKD46 were then electroporated with the respective targeting fragments. Following homologous recombination and antibiotic selection, candidate clones were screened by colony PCR using four independent primer pairs. All mutants (*E. coli* Δ*ybhS, E. coli* Δ*ybhR*, *E. coli* Δ*ybhSR, and E. coli* Δ*ybhFSR*) yielded PCR products distinct from those of wild-type *E. coli* K-12 (Figures 2A–D). Representative isolates were sequenced (Sangon Biotech, Shanghai), and correctly edited strains were stored at −80 °C for subsequent analyses.

**Figure 2:**
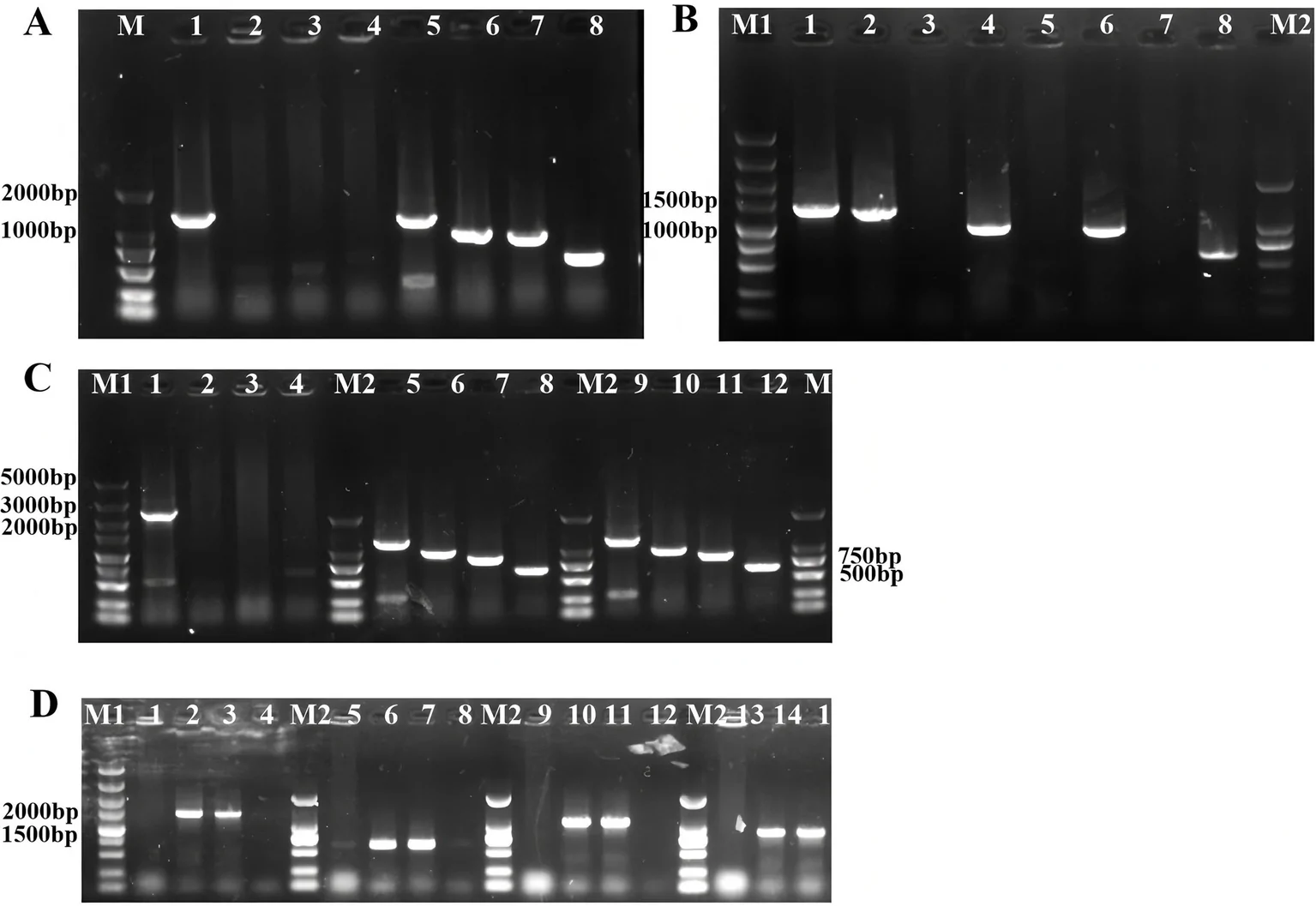
Colony PCR verification of the gene deletion mutants using four independent primer pairs. **(A)** Δ*ybhS* mutant. Lanes 1–4: wild-type *E. coli* K-12; lanes 5–8: Δ*ybhS* positive clone. Primer pairs and expected amplicon sizes: lanes 1 and 5, T-*ybhS* F/T-*ybhS* R (1,371 bp for WT, 1295 bp for Δ*ybhS*); lanes 2 and 6, T-*ybhS* F/*Cm*-R (no band for WT, 1341 bp for Δ*ybhS*); lanes 3 and 7, *Cm*-F/T-*ybhS* R (no band for WT, 1358 bp for Δ*ybhS*); lanes 4 and 8, *Cm*-F/*Cm*-R (no band for WT, 647 bp for Δ*ybhS*). **(B)** Δ*ybhR* mutant. Lanes 1, 3, 5, 7: wild-type *E. coli* K-12; lanes 2, 4, 6, 8: Δ*ybhR* positive clone. Primer pairs and expected amplicon sizes: lanes 1 and 2, T-*ybhR* F/T-*ybhR* R (1,378 bp for WT, 1381 bp for Δ*ybhR*); lanes 3 and 4, T-*ybhR* F/Cm-R (no band for WT, 1072 bp for Δ*ybhR*); lanes 5 and 6, *Cm*-F/T-*ybhR* R (no band for WT, 943 bp for Δ*ybhR*); lanes 7 and 8, Cm-F/Cm-R (no band for WT, 628 bp for ΔybhR). **(C)** Δ*ybhSR* mutant. Sample lanes 1–4: wild-type *E. coli* K-12; sample lanes 5–8: ΔybhSR1 positive clone; sample lanes 9–12: Δ*ybhSR*2 positive clone. Primer pairs and expected amplicon sizes: lanes 1, 5 and 9, T-*ybhS* F/T-*ybhR* R (2,405 bp for WT, 1268 bp for Δ*ybhSR*); lanes 2, 6 and 10, T-*ybhS* F/*Cm*-R (no band for WT, 952 bp for Δ*ybhSR*); lanes 3, 7 and 11, *Cm*-F/T-*ybhR* R (no band for WT, 843 bp for Δ*ybhSR*); lanes 4, 8 and 12, *Cm*-F/*Cm*-R (no band for WT, 628 bp for Δ*ybhSR*). **(D)** Δ*ybhFSR* mutant. Sample lanes 1, 5, 9, 13: wild-type *E. coli* K-12; sample lanes 2, 6, 10, 14: Δ*ybhFSR*1 positive clone; sample lanes 3, 7, 11, 15: Δ*ybhFSR*2 positive clone; sample lanes 4, 8, 12, 16: blank control (water as template). Primer pairs and expected amplicon sizes: lanes 1–4, T-*ybhF* F/T-*ybhR* R (4,607 bp for WT, 1632 bp for Δ*ybhFSR*, no band for blank); lanes 5–8, *Cm*-F/*Cm*-R (no band for WT, 647 bp for Δ*ybhFSR*, no band for blank); lanes 9–12, T-*ybhF* F/Cm-R (no band for WT, 1322 bp for Δ*ybhFSR*, no band for blank); lanes 13–16, *Cm*-F/T-*ybhR* R (no band for WT, 955 bp for Δ*ybhFSR*, no band for blank). In panels **(C,D)**, lanes labeled M1 and M2 are DNA markers (M1, DL5000; M2, DL2000) and are not sample lanes. All PCR products were analyzed by 1% agarose gel electrophoresis. Each sample lane represents an independent colony PCR reaction, and the experiment was repeated three times with similar results (*n* = 3 independent replicates).

To exclude potential growth defects, wild-type and mutant strains were cultivated in LB medium, and optical density (OD₆₀₀) was monitored hourly over 12 h. All deletion strains reached final culture densities comparable to that of the wild type after 9–12 h of incubation, although significant differences in OD₆₀₀ were observed during the exponential phase (4–8 h) (Figure 3). These results confirm the successful construction of the mutants. Importantly, the loss of *ybhS* and/or *ybhR* does not substantially impair overall growth capacity, even though modest growth-rate differences during exponential phase were detectable.

**Figure 3:**
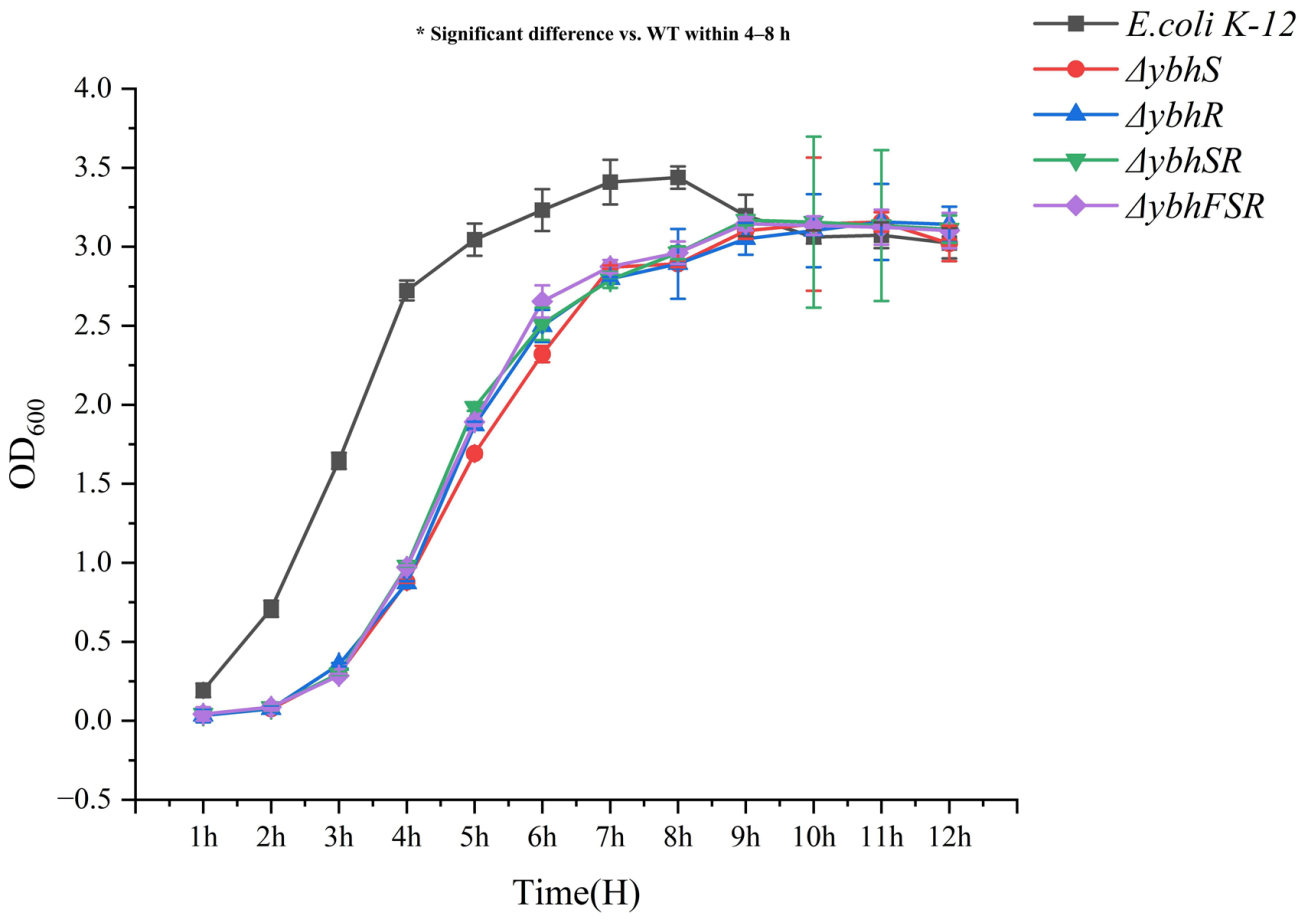
Growth curves of *E. coli* K-12 WT and mutant strains (Δ*ybhS*, Δ*ybhR*, Δ*ybhSR*, and Δ*ybhFSR*) in LB medium. The optical density at 600 nm (OD₆₀₀) was measured every 1 h over a 12-h period. Data are mean ± SD (*n* = 3 biological replicates, each with three technical replicates). *p < 0.05, **p < 0.01, ***p < 0.001 vs. WT (two-way repeated-measures ANOVA with strain as the between-subject factor and time as the within-subject factor, followed by Tukey’s *post hoc* test; sphericity was assessed and Greenhouse–Geisser correction applied when required). Mutants showed significantly lower OD₆₀₀ at 4–8 h but reached comparable final OD₆₀₀ values by stationary phase (9–12 h, *p* > 0.05); given the limited sample size (*n* = 3), this should not be interpreted as evidence of equivalence.

### 3.2 Dose-dependent transcriptional upregulation of the *ybhFSR* operon under TC stress

Wild-type *E. coli* K-12 was exposed to increasing TC concentrations (0–25 μg/mL), and transcript levels of *ybhF*, *ybhS*, *ybhR*, and *acrB* were quantified by qRT-PCR (Figures 4A–D). All four genes were upregulated in a dose-dependent manner. At 25 μg/mL TC, *ybhR* showed the strongest induction (~11.5-fold), followed by *ybhS* (~10.4-fold), *ybhF* (~5.8-fold), and *acrB* (~4.5-fold) (all *p* < 0.001). We further observed significantly higher transcript abundance of *ybhR* relative to *ybhS* at every tested TC concentration (*p* < 0.01), revealing unequal transcriptional activation of the two TMD genes within a single operon.

**Figure 4:**
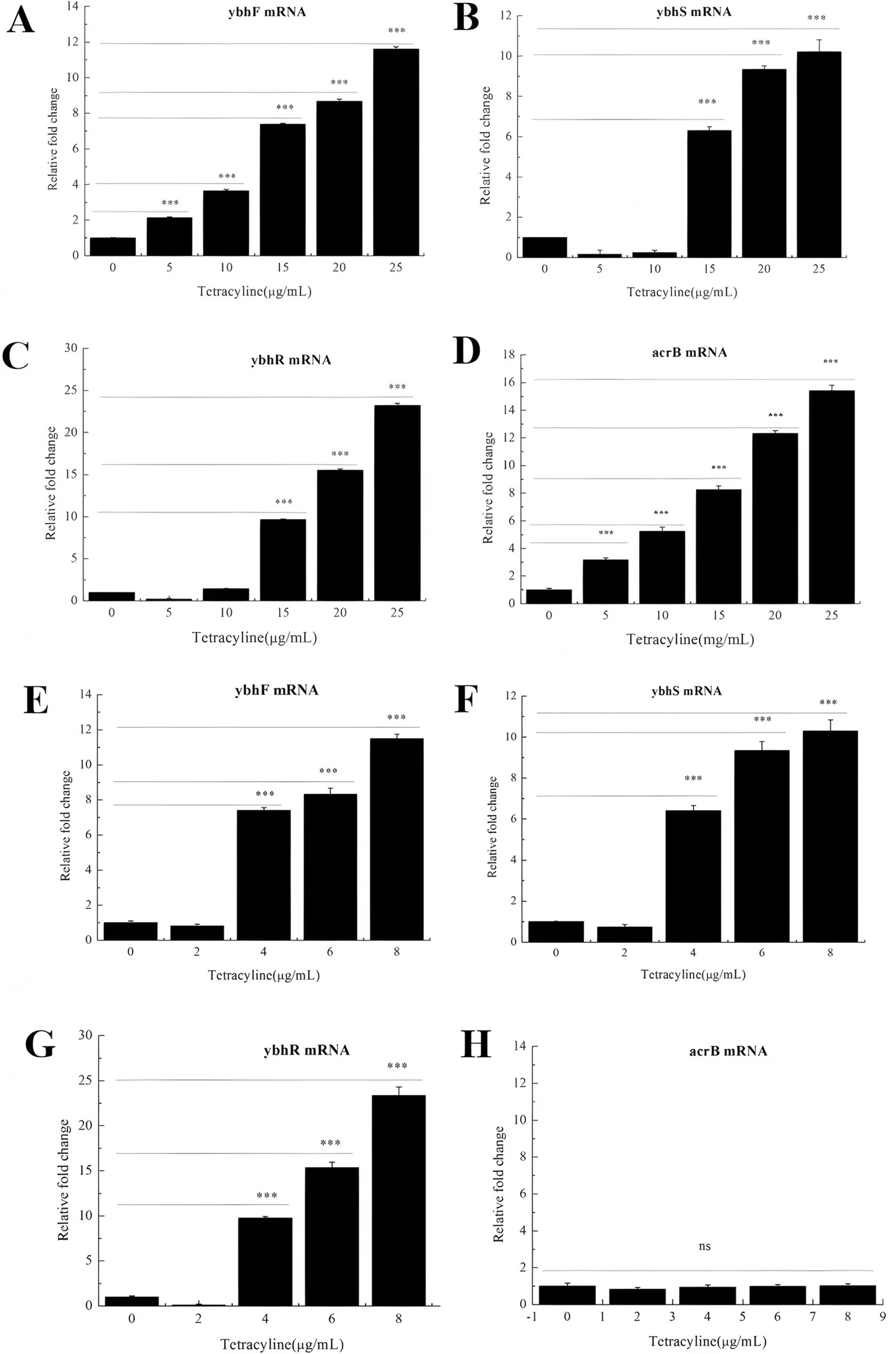
Relative mRNA levels of *ybhF*, *ybhS*, *ybhR*, and *acrB* in wild-type *E. coli* K-12 and the Δ*acrB* deletion mutant under increasing concentrations of tetracycline (TC). **(A–D)** Dose-dependent expression of *ybhF*, *ybhS*, *ybhR*, and *acrB* in wild-type cells induced with 0, 5, 10, 15, 20, and 25 μg/mL TC. **(E–H)** Corresponding transcript levels of *ybhF*, *ybhS*, *ybhR*, and *acrB* in the Δ*acrB* mutant induced with 0, 2, 4, 6, and 8 μg/mL TC. Expression values were normalized using the 2^−ΔΔCt^ method. Data represent mean ± SD from three independent biological replicates (*n* = 3). Statistical significance was determined by one-way ANOVA followed by Dunnett’s *post hoc* test for multiple comparisons against the 0 μg/mL control. *p < 0.05, **p < 0.01, ***p < 0.001; ns, not significant.

We performed parallel TC induction experiments using the Δ*acrB* mutant, which lacks the major RND (Resistance-Nodulation-Division) efflux pump, to assess whether AcrAB depletion alters *ybhFSR* transcriptional profiles (0–8 μg/mL TC; Figures 4E–H). Consistent with the strain genotype, no *acrB* mRNA was detectable. Even without functional AcrAB-TolC, increasing TC levels elevated transcript levels of *ybhF*, *ybhS* and *ybhR*. Quantitative comparison further revealed markedly higher induction levels in the Δ*acrB* mutant than wild-type cells. At 8 μg/mL TC, transcript fold changes reached 23.8 for *ybhR*, 11.6 for *ybhF* and 10.2 for *ybhS* (all *p* < 0.001). These transcriptional shifts show that removing AcrAB-TolC elevates *ybhFSR* expression to a marked degree, pointing to its auxiliary detoxification function when the primary efflux route is impaired.

### 3.3 Differential susceptibility of *ybh* mutants to TC-class antibiotics

Disk diffusion assays revealed that deletion of *ybhF* increased inhibition zones relative to wild type (Table 2). The Δ*ybhS* and Δ*ybhR* single mutants displayed inhibition zones essentially indistinguishable from each other and only marginally different from the wild type under these conditions, whereas the Δ*ybhSR* and Δ*ybhFSR* mutants showed clearer increases (Figure 5).

**Table 2:** Inhibition zone diameters of tested strains against four tetracycline-class antibiotics (Mean ± SD, mm).

Drugs	WT	pET28a-*ybhF*	Δ*ybhF*	Δ*ybhS*	Δ*ybhR*	Δ*ybhSR*	Δ*ybhFSR*	Δ*acrB*	Δ*acrB*Δ*ybhF*
TC	21 ± 0.1	19 ± 0.1	23 ± 0.2	21 ± 0.1	21 ± 0.1	23 ± 0.1	25 ± 0.1	24 ± 0.2	29 ± 0.2
OTC	18.5 ± 0.1	12 ± 0.2	21 ± 0.2	19 ± 0.1	19 ± 0.1	19 ± 0.1	21 ± 0.1	20.5 ± 0.1	24 ± 0.1
CTC	19 ± 0.1	16 ± 0.1	22 ± 0.1	20 ± 0.2	20 ± 0.1	21.5 ± 0.2	22 ± 0.2	24 ± 0.1	28.5 ± 0.1
DOX	17.5 ± 0.1	16.5 ± 0.1	21 ± 0.1	18 ± 0.1	18 ± 0.1	22 ± 0.1	24 ± 0.1	24.5 ± 0.1	28 ± 0.1

**Figure 5:**
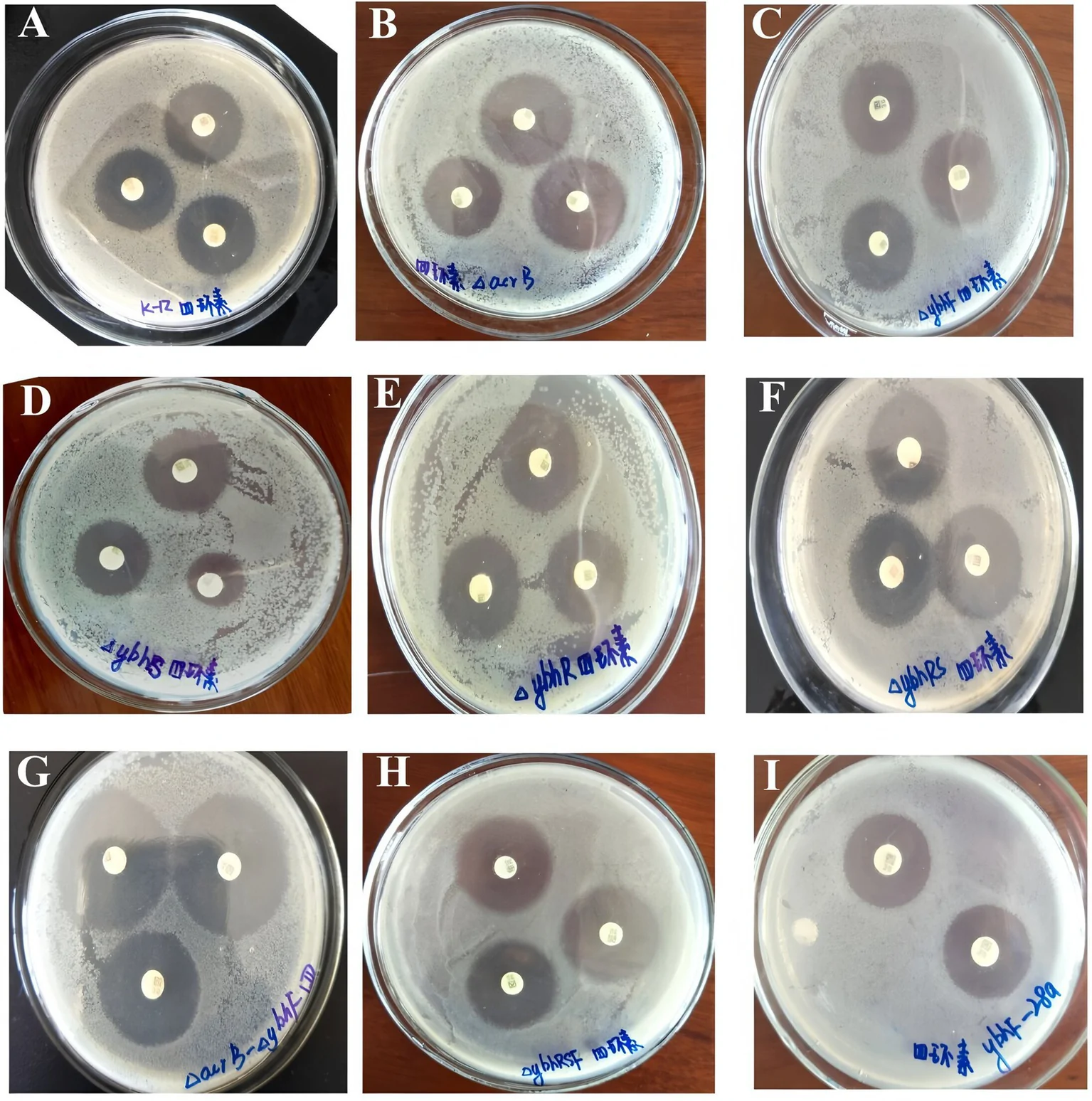
Disk diffusion assay evaluating tetracycline (TC) susceptibility in wild-type *E. coli* K-12 and various mutant strains. Representative images of inhibition zones formed around TC disks. Tested strains are labeled on the plates: **(A)** wild-type *E. coli* K-12, **(B)** Δ*acrB* mutant, **(C)** Δ*ybhF* mutant, **(D)** Δ*ybhS* mutant, **(E)** Δ*ybhR* mutant, **(F)** Δ*ybhRS* mutant, **(G)** Δ*acrB*-Δ*ybhF* double mutant, **(H)** Δ*ybhRSF* triple mutant, and **(I)** Δ*ybhF* mutant carrying the complementation plasmid pET28a-*ybhF*. All diffusion tests were incubated at 35 °C for 20 h on MH agar. The diameter of the growth inhibition zones (ZOI) directly reflects strain susceptibility to tetracycline. The images are representative of three independent biological replicates (*n* = 3). As this figure presents qualitative image data, the quantitative analysis is presented in Figure 6.

Combined deletions further delineated subunit contributions. The Δ*ybhSR* double mutant yielded a zone of 23 mm, marginally larger than that of Δ*ybhR,* whereas the Δ*ybhFSR* triple mutant produced a 25 mm zone. These data indicate that loss of *ybhR* impairs efflux and that additional deletion of *ybhS* does not further enhance susceptibility. Complete inactivation of the transporter, accompanied by maximal susceptibility, required deletion of *ybhF* together with both transmembrane subunit genes.

To assess relationship with the major RND efflux system, we examined Δ*acrB* backgrounds. The Δ*acrB* single mutant exhibited a 24 mm zone (*p* < 0.01 vs. wild type), confirming its role in intrinsic resistance. The Δ*acrB*Δ*ybhF* double mutant displayed the largest zone (29 mm), significantly exceeding either single mutant (*p* < 0.001). This synergistic effect suggests that YbhFSR and AcrAB-TolC mediate TC resistance through independent pathways.

The OTC, CTC, and DOX yielded analogous results (Figure 6). The ybhFSR deletion series consistently enhanced susceptibility. The pET28a-*ybhF* overexpression strain tended to produce smaller inhibition zones than the wild type across all four tetracyclines (e.g., 19 vs. 21 mm for TC and 12 vs. 18.5 mm for OTC; Table 2), consistent with a modest increase in efflux capacity upon plasmid-driven *ybhF* expression.

**Figure 6:**
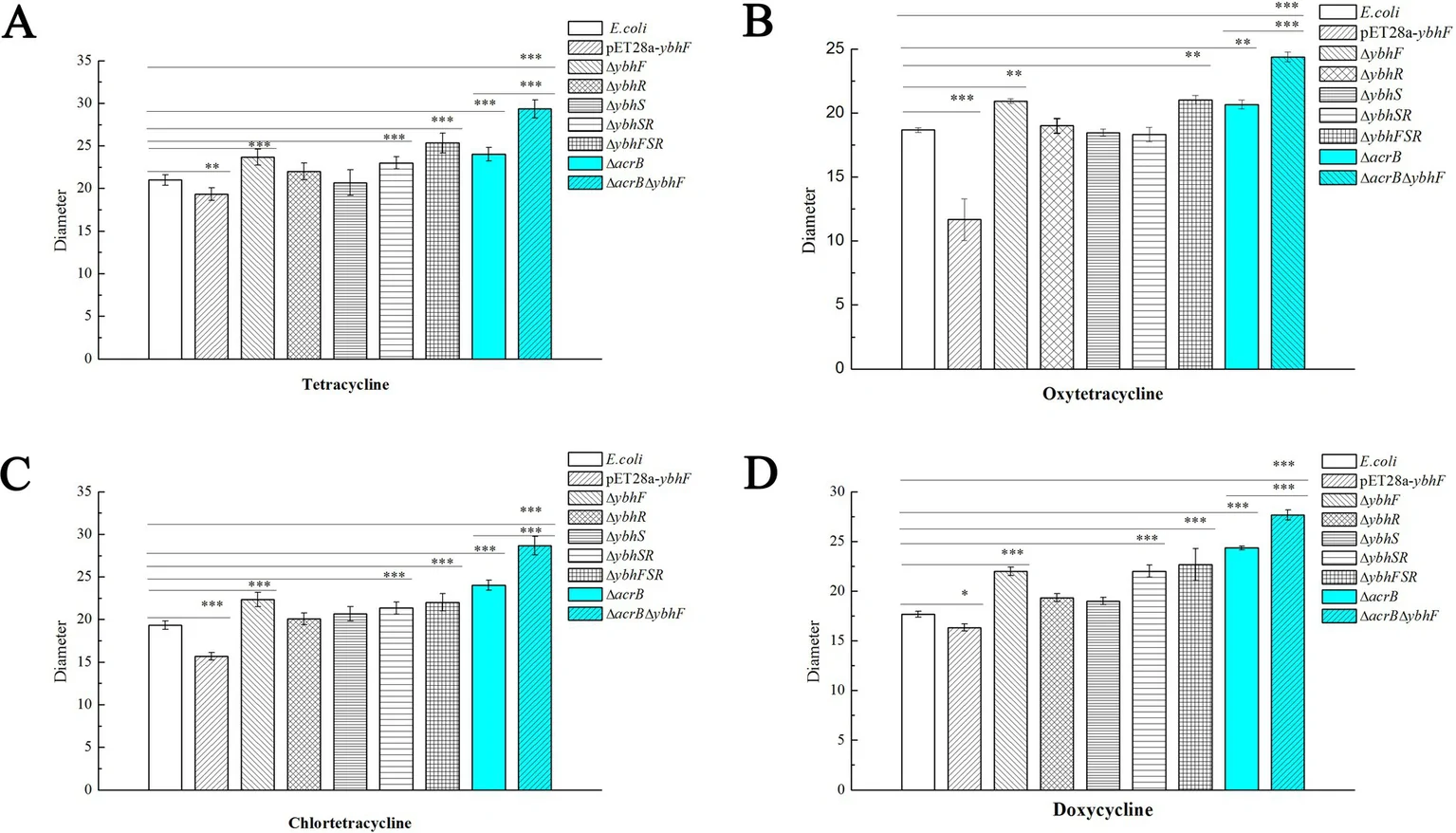
Growth inhibition zone (ZOI) diameters from disk diffusion assays for wild-type *E. coli* K-12 and mutant strains against four tetracycline-family antibiotics. **(A)** Tetracycline (TC); **(B)** oxytetracycline (OTC); **(C)** chlortetracycline (CTC); **(D)** doxycycline (DOX). Assays were incubated on Mueller-Hinton agar at 35 °C for 20 h. Data are presented as mean ± SD from three independent biological replicates (*n* = 3). Statistical analysis was performed using one-way ANOVA followed by Tukey’s *post hoc* test for multiple comparisons. Significant differences versus the wild-type strain are indicated as *p < 0.05, **p < 0.01, ***p < 0.001.

Because disk diffusion did not resolve the functional difference between Δ*ybhS* and Δ*ybhR*, broth microdilution MIC determination was used to quantify their respective contributions.

### 3.4 Distinct contributions of *ybhS* and *ybhR* deletions to TC susceptibility

To compare the functional roles of the two TMD subunits, we determined MICs of a series of deletion mutants by broth microdilution (Table 3).

**Table 3:** MIC of the tested antimicrobial drugs.

Drugs	Minimum inhibitory concentrations (μg/mL)				
	*E.coli*	∆*ybhS*	∆*ybhR*	∆*ybhSR*	∆*ybhFSR*
TC	1	1	0.5	0.5	0.5
OTC	32	32	16	16	16
CTC	64	64	1	1	1
Dox	32	32	1	1	1
Ethidium	256	256	128	128	128
Hoechst 33342	128	128	64	64	64
Cefoperazone	1	1	1	1	1
Cefazolin	64	64	64	32	32
Streptomycin	>256	>256	>256	>256	>256
Ampicillin	8	8	8	8	8
Roxithromycin	>256	>256	>256	>256	>256
Chloramphenicol	32	32	32	32	32
Rifampicin	16	16	16	16	16
Norfloxacin	2	2	2	2	2
Deoxycholate	>256	>256	>256	>256	>256
Sodium cholate	>256	>256	>256	>256	>256
Ofloxacin	0.125	0.125	0.125	0.125	0.125
Doxorubicin	4	4	4	4	4
Daunorubicin	64	64	64	64	64
Acridine flavin	0.5	0.5	0.5	0.5	0.5
Quinine	>256	>256	>256	>256	>256

Minimum inhibitory concentrations (MICs) of the tested antimicrobial drugs. MICs are expressed in μg/mL. “>256” indicates that no visible growth inhibition was observed at the highest concentration tested (256 μg/mL).

The Δ*ybhR* mutant exhibited increased susceptibility to all four tetracyclines. Compared with the wild type, MICs of OTC and TC were 2-fold lower, whereas those of CTC and DOX were reduced 64-fold and 32-fold, respectively. In contrast, the Δ*ybhS* mutant showed no change in MIC to any tested TC, indicating that loss of YbhS does not impair efflux at the cellular level.

The MIC values of the Δ*ybhSR* double mutant and Δ*ybhFSR* triple mutant were indistinguishable from those of the Δ*ybhR* single mutant. Additional deletion of *ybhS* did not further reduce MICs, nor was any additive effect on susceptibility detected. Moreover, MIC testing against approximately 20 unrelated antimicrobials revealed no differences between any *ybh* mutant and the wild type, indicating that YbhFSR exhibits narrow substrate specificity. MIC changes were similar across all four tetracyclines, with only Δ*ybhR* mutants showing reduced MICs.

### 3.5 Fluorometric assay of TC efflux activity

To directly evaluate the functional contributions of YbhS and YbhR to TC efflux, we measured real-time TC export into the extracellular compartment using a multi-mode microplate reader. Wild-type K-12 and mutant strains (Δ*ybhS*, Δ*ybhR*, Δ*ybhSR*, and Δ*ybhFSR*) were assayed at equal cell density (OD₆₀₀ = 0.8) with an identical initial TC concentration of 100 μg/mL. Time-dependent extracellular fluorescence intensity is presented in Figure 7.

**Figure 7:**
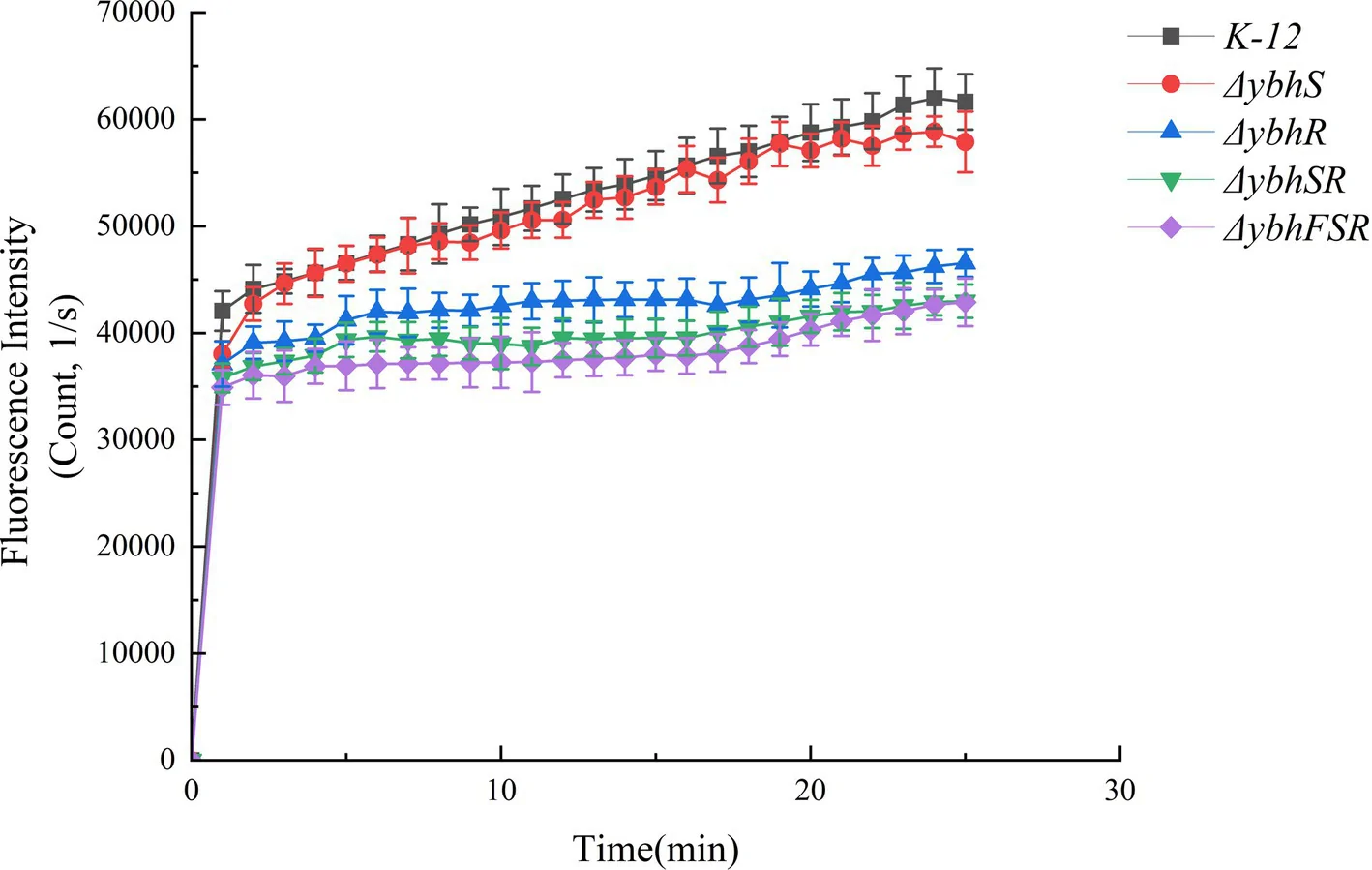
Time-course tetracycline (TC) efflux profiles of wild-type *E. coli* K-12 and Δ*ybh* mutant strains. Cells were pre-loaded with TC, and the fluorescence intensity was monitored by spectrofluorometry over 25 min. An increase in fluorescence intensity was interpreted as greater apparent TC efflux activity. Data are presented as mean ± SD from three independent biological replicates (*n* = 3). Statistical analysis was performed using two-way ANOVA with Tukey’s *post hoc* test for multiple comparisons. At the 25-min time point, the efflux activities of Δ*ybhR*, Δ*ybhSR*, and Δ*ybhFSR* were significantly lower than that of the WT strain (*p* < 0.001), whereas no significant difference was observed between Δ*ybhS* and the WT strain (*p* > 0.05).

Upon TC addition, extracellular fluorescence rose steadily in wild-type K-12 cells. Efflux kinetics of the Δ*ybhS* mutant closely followed the wild-type profile, with only a slight deviation after 20 min. By contrast, TC efflux was impaired in the Δ*ybhR* mutant, which yielded a 24.2% reduction in extracellular fluorescence signal at 25 min relative to wild-type cells. The Δ*ybhSR* and Δ*ybhFSR* strains exhibited efflux defects similar to the Δ*ybhR* single-mutant phenotype.

### 3.6 Cross-species conservation and sequence divergence of YbhS and YbhR

To assess evolutionary conservation differences between YbhS and YbhR, we performed BLAST comparisons on orthologs from seven *Enterobacteriaceae* genera (Table 4). YbhR showed higher mean sequence identity than YbhS in five of the seven genera (96.20% vs. 93.89%, a difference of 2.31%), with the largest difference in *Citrobacter* (3.29%). The sole exception was *Serratia*, where YbhS was more highly conserved (78.51% vs. 72.28%, a difference of −6.23%). Full-length YbhS and YbhR share only 24.9% identity and 50.0% similarity.

**Table 4:** Cross-species conservation analysis of YbhR and YbhS.

Genus	Species	YbhR identity (%)	YbhS identity (%)	Difference (R − S)
*Escherichia*	*E. coli* O157: H7	100.00	100.00	0.00
*Shigella*	*S. flexneri*	100.00	99.47	+0.53
*Enterobacter*	*E. cancerogenus*	95.65	93.90	+1.75
*Salmonella*	*S. enterica*	95.38	93.09	+2.29
*Klebsiella*	*K. pneumoniae*	92.66	91.51	+1.15
*Citrobacter*	*C. tevivensis*	93.48	90.19	+3.29
*Serratia*	*Serratia* spp.	72.28	78.51	−6.23

Across the seven genera, YbhR identity exceeded that of YbhS in five, was lower in one (Serratia, −6.23%), and was identical in Escherichia. Excluding the tie, a Wilcoxon signed-rank test gave *p* = 0.0625; Excluding the tie (*Escherichia*), six paired values remained (5 positive, 1 negative); a Wilcoxon signed-rank test gave *p* = 0.0625. a paired t-test across all seven genera gave *t* = 0.337 (df = 6), *p* = 0.75. At this sample size both tests have limited power, so we report the direction of the difference without claiming significance. Serratia is the sole exception, and a single genus cannot distinguish relaxed purifying selection on YbhR in this lineage from a shift in substrate range or gene retention. Resolving this requires biochemical or structural data; we treat it as an open question rather than evidence of functional divergence.

We next compared aromatic amino acid composition between the two proteins. In transmembrane helices, Trp accounted for a 2.1-fold higher fraction of aromatic residues in YbhR than in YbhS, whereas phenylalanine dominated in YbhS (72.2% of its transmembrane aromatic residues). In non-transmembrane regions, YbhR was enriched in tyrosine and tryptophan relative to YbhS (11.81% vs. 7.95%), with Tyr and Trp accounting for most of this difference. Together, these compositional differences raise the possibility that YbhR plays a primary role in substrate engagement and allosteric coupling.

## 4 Discussion

Our data reveal that the two TMD subunits of the YbhFSR transporter, YbhS and YbhR, are functionally asymmetric during TC efflux. These findings extend current understanding of ABC transporter subunit division beyond Gram-positive heterodimeric systems to a Gram-negative transporter with a distinctive “homologous NBD + heterologous TMD” architecture.—fused yet functionally distinct NBDs paired with heterodimeric TMDs—functional asymmetry can be dissected across both structural layers, offering a simplified model for studying subunit specialization in ABC transporters. Furthermore, our transcriptional data demonstrate that *ybhFSR* is strongly induced upon TC challenge and becomes hyperactivated when the major AcrAB-TolC pump is deleted, indicating that YbhFSR serves as a compensatory efflux pathway.

### 4.1 Transcriptional response of YbhFSR as a compensatory pathway for AcrAB-TolC

Multidrug-resistant *E. coli* is a growing public health threat. Overexpression of efflux pumps including AcrAB-TolC is a major contributor (Kobylka et al., 2020; Bahaj et al., 2025). Bacterial genomes encode dozens of efflux pumps, which are not expressed simultaneously or at equal levels. In *E. coli*, the RND-family AcrAB-TolC system functions as the primary pump with the highest expression and broadest substrate spectrum, establishing basal intrinsic antibiotic resistance (Li et al., 2015). When AcrAB-TolC becomes non-functional due to drug saturation or gene mutation, bacteria can rapidly activate regulatory networks to induce compensatory expression of secondary efflux pumps (Cudkowicz and Schuldiner, 2019). This redundancy within the bacterial effluxome is well documented. In evolutionary terms, adaptive mutations that drive sustained expression of alternative pathways are termed compensatory evolution (Cho and Misra, 2021). Consistent with this, laboratory-evolved or mutant strains lacking AcrAB frequently upregulate genes including *mdfA*, *emrE*, and the *ybhFSR* operon characterized here (Nishino and Yamaguchi, 2001; Cho and Misra, 2021). However, the precise regulators governing this compensatory upregulation remain to be identified.

Strong transcriptional activation of the *ybhFSR* operon observed in the Δ*acrB* background (Figures 3E–H) directly reflects this compensatory pattern. qRT-PCR data showed transcription of *ybhR* and *ybhS* rises in a dose-dependent manner with increasing TC concentrations in wild-type cells, and this induction is markedly amplified in the Δ*acrB* strain. At 8 μg/mL tetracycline, *ybhR* expression increased approximately 23.8-fold and *ybhS* around 10.2-fold. Inactivation of AcrAB-TolC can drive compensatory expression of secondary efflux pumps via global regulators including RamA and MarA (Blair et al., 2014; Zhang et al., 2018). To search for regulatory proteins acting on the *ybhFSR* operon, we performed DNA pull-down using the *ybhF* promoter fragment as bait, followed by LC–MS/MS. Four stress-responsive transcriptional regulators were enriched at this promoter: ArcA (FC = 238.28), OmpR (64.77), CpxR (17.39) and PhoP (9.67). Neither RamA nor MarA, nor the BaeSR system, was detected. Notably, CecR, another characterized antibiotic- responsive transcriptional regulator in E. coli, was also not identified in our pull-down assay (Yamanaka et al., 2016).

Because a single pull-down cannot exclude interactions occurring under other growth conditions, these negative results do not place *ybhFSR* outside the broader AcrAB-associated regulatory network; they suggest only that RamA- or MarA-dependent regulation is unlikely to dominate this operon.

Of the four, CpxR, OmpR and PhoP are the most tractable candidates for validation: their cognate systems sense envelope stress, osmotic stress and Mg^2+^ limitation, respectively, and all three conditions are expected to accompany intracellular tetracycline accumulation once the major efflux route is impaired. ArcA showed the strongest enrichment, but its fold change is ~3.7-fold that of the next-ranked protein, a gap that may partly reflect high cellular abundance rather than promoter-specific binding; independent verification is therefore required. For each candidate, EMSA or ChIP-qPCR combined with promoter-reporter assays will be needed to establish direct regulation of *ybhFSR*.

Importantly, we detected consistent, statistically significant differences in induction fold changes between *ybhR* and *ybhS* in both wild-type and Δ*acrB* backgrounds. Such transcriptional divergence suggests that subunits encoded within the same operon are not strictly co-transcribed at proportional levels. This could reflect internal promoter activity or differential mRNA stability, though distinguishing between these possibilities will require promoter activity reporters and mRNA decay assays.

### 4.2 Functional non-equivalence of transmembrane subunits YbhS and YbhR in TC efflux

The TMD subunits YbhS and YbhR form a functionally asymmetric pair within YbhFSR. YbhR appears to dominate tetracycline efflux, whereas YbhS contributes little—if at all—to substrate extrusion. This pattern of unequal subunit partitioning is consistent with functional subfunctionalization observed in heterodimeric ABC efflux systems of Gram-positive bacteria (Timachi et al., 2017; Tang et al., 2023).

Unlike canonical Gram-positive ABC transporters with discrete NBD subunits, YbhFSR features a single polypeptide harboring two fused NBD domains. Sequence divergence between TMD paralogs is a well-established determinant of differential substrate affinity and interdomain allosteric coupling, underpinning functional asymmetry in heterodimeric ABC transporters (Zolnerciks et al., 2011) and aligning with the classical alternating-access model. Despite its atypical fused-NBD architecture, TMD sequence divergence between YbhS and YbhR is associated with functional asymmetry, supporting a central role for YbhR in tetracycline engagement and ATP-driven conformational signaling, whereas YbhS likely acts as a structural auxiliary component.

Notably, functional asymmetry in YbhFSR extends beyond the TMD pair. The tandem NBD domains within YbhF also exhibit intrinsic inequality: NBD1 retains canonical ABC catalytic motifs, whereas NBD2 harbors multiple catalytically disabling substitutions, paralleling the asymmetric NBD organization of TM287/288 (Timachi et al., 2017). This domain divergence suggests a dominant catalytic role for NBD1 and an auxiliary role for NBD2. Collectively, layered asymmetry at both NBD and TMD levels concentrates core transport function in YbhR, representing a distinct variant of functional specialization among bacterial ABC exporters. This architecture thus provides a valuable model for dissecting how multilevel functional asymmetry is coordinated across transporter domains.

Current interpretations are based on cellular phenotypic evidence and do not exclude indirect structural or regulatory roles for YbhS, as reported for accessory subunits in other ABC systems (Biemans-Oldehinkel et al., 2006; Koch et al., 2006; Tang et al., 2023; Tang et al., 2025). Although dispensable for TC efflux, YbhS may facilitate complex assembly, membrane targeting, or conformational dynamics of the transporter holocomplex.

A related issue is the discrepancy between disk diffusion and MIC results. Disk diffusion failed to detect the YbhS/YbhR asymmetry seen in MIC assays, likely because zone diameter integrates multiple variables—drug diffusion, agar composition, stability, and growth rate—and does not scale linearly with MIC. For TC and OTC, ΔybhR showed only a 2-fold MIC reduction, below the detection limit of disk diffusion; accordingly, both mutants produced zones indistinguishable from WT. For CTC and DOX, MIC reductions were larger (64-fold and 32-fold), yet zone-diameter differences remained subtle (20 vs. 19 mm for CTC; 18 vs. 17.5 mm for DOX). This probably reflects the non-linear, compressed relationship between zone size and MIC, compounded by drug-specific differences in how TC, OTC, CTC, and DOX diffuse and persist in MH agar. We therefore relied primarily on MIC data; disk diffusion was used for qualitative confirmation only.

### 4.3 Divergent evolutionary conservation reveals general rules governing functional differentiation of TMD subunits

Cross-species sequence alignments show that YbhR exhibits higher sequence conservation than YbhS in five of seven core Enterobacteriaceae genera, although this difference did not reach statistical significance in our paired comparisons (exact Wilcoxon signed-rank test, *p* = 0.0625; paired *t*-test, *p* = 0.75). Meanwhile, full-length sequence identity between the two subunits reaches only 24.9%, a range associated with substantial functional divergence among homologous proteins. These two datasets together reveal a striking discordance: YbhR is highly conserved in interspecies comparisons, yet diverges greatly from YbhS within the same organism. Such asymmetry may reflect a broader principle governing TMD subunit evolution in heterodimeric ABC transporters: the leading subunit is subject to stronger purifying selection, whereas the accessory subunit tolerates greater sequence variation.

#### 4.3.1 The leading subunit shows a trend toward stronger purifying selection

In the alternating access model, the leading subunit in heterodimeric ABC transporters typically performs three essential functions: forming the substrate-binding pocket, receiving allosteric signals from NBDs, and transmitting ATP-driven conformational changes to the substrate site (Davidson et al., 2008; Hollenstein et al., 2007; Zolnerciks et al., 2011). Key structural elements supporting these functions — aromatic residues lining the substrate pocket and critical residues at the TMD–NBD interface — must maintain precise geometry for efficient energy transfer. Mutations at these sites can abolish transporter activity.

Recent work on the bacterial multidrug ABC transporter BmrA confirmed that a conserved tryptophan-centered residue cluster at the NBD–TMD interface acts as a hub for bidirectional signal transmission. Mutations within this hub uncouple ATP hydrolysis from transport activity (Pérez Carrillo et al., 2025). This finding provides direct evidence that coupling helices require considerable structural rigidity to sustain efficient NBD–TMD coupling.

Although the conservation difference did not reach statistical significance in our seven-genus comparison, the consistent direction of the effect (YbhR more conserved in five of seven genera) suggests that purifying selection may act more stringently on YbhR. This inference is consistent with known patterns of heterodimeric ABC transporters. Cryo-EM structures of BmrCD show that the substrate Hoechst-33342 primarily interacts with transmembrane helices of BmrC, while BmrD contributes mainly to conformational stabilization (Thaker et al., 2022; Tang et al., 2025). Accessory subunits face fewer functional constraints and therefore tolerate more sequence variation. Domain-swapping experiments further demonstrate that certain domains of accessory subunits can be replaced without loss of transport activity (Daumke and Knittler, 2001).

One notable exception is Serratia, where YbhS is 6.23% more conserved than YbhR, opposite to the pattern in the other six genera. This could reflect lineage-specific functional divergence of the TMD subunits, or genus-specific differences in substrate preference and ecological niche. Our current dataset cannot distinguish between these possibilities, and resolving this will require functional characterization of YbhFSR in *Serratia*.

#### 4.3.2 Sequence divergence as an evolutionary signature of functional differentiation depth

The 24.9% full-length identity between YbhS and YbhR falls within the range of canonical heterodimeric ABC transporters, including *B. subtilis* BmrC/BmrD (roughly 22–25%) and the thermophilic TM287/288 (approximately 27%) (Timachi et al., 2017; Tang et al., 2023). Sequence divergence often correlates with functional specialization (Thomas et al., 2020). In BmrCD, sequence identity of 22–25% is sufficient to maintain global folding and co-assembly, while substantial sequence divergence provides a structural foundation for functional specialization (Rees et al., 2009).

Notably, the architecture of YbhFSR — two NBD domains encoded within a single YbhF polypeptide and heterodimeric TMDs — offers a unique viewpoint for understanding how intrinsic TMD divergence relates to functional asymmetry. Unlike BmrCD and TM287/288, where functional asymmetry partly originates from differences between two separate NBD subunits, YbhF contains two fused yet intrinsically asymmetric NBDs, with NBD1 retaining the canonical catalytic motifs and NBD2 carrying the degenerate LSGGM → LRGRA substitution (Feng et al., 2026). Even under this condition, pronounced functional divergence has evolved between YbhS and YbhR. This raises the possibility that TMD sequence divergence alone can drive functional asymmetry. Srikant et al. (2020) conducted random mutagenesis on the TMD of fungal ABC transporter Ste6. Their experimental evolution data support this idea, showing that TMD sequence plasticity can drive differentiation of substrate specificity.

#### 4.3.3 Differences in aromatic residue distribution provide a structural basis for substrate recognition

YbhR contains 2.1-fold more tryptophan within its transmembrane region than YbhS. Tryptophan mediates stronger π–π stacking with aromatic substrates than phenylalanine (Samanta et al., 1999; Xu et al., 2020). We hypothesize that the relative enrichment of Trp in the transmembrane domain of YbhR could enhance affinity toward tetracycline substrates through π–π stacking, but this prediction remains to be tested directly. Cryo-EM structures of BmrCD reveal marked asymmetry in substrate contacts between subunits; BmrC contributes most aromatic residues participating in ligand binding (Thaker et al., 2022; Tang et al., 2023).

YbhR also contains more Tyr and Trp in non-transmembrane segments, including coupling helices and intracellular regulatory regions. Coupling helices mediate allosteric signal transduction between NBDs and TMDs and undergo conformational rearrangement upon substrate binding and ATP hydrolysis (Novischi et al., 2024). A Trp-centered residue cluster in BmrA has been identified as a bidirectional communication hub linking NBD and TMD domains (Pérez Carrillo et al., 2025). We speculate that enriched Tyr and Trp in non-transmembrane regions of YbhR might constitute an analogous allosteric signal-receiving interface, potentially allowing ATP hydrolysis-triggered conformational changes to propagate preferentially through YbhR toward the substrate-binding pocket. This prediction will require structural and mutational confirmation.

#### 4.3.4 Structural implications

Together, these three lines of evidence — the trend toward higher interspecies conservation of YbhR, extensive sequence divergence from YbhS, and specific enrichment of Trp/Tyr residues — are consistent with, but do not yet establish, YbhR functioning as the primary TMD subunit for substrate recognition and energetic coupling, while YbhS serves predominantly structural roles. This functional asymmetry is not unique to YbhFSR; it reflects a recurring theme in heterodimeric ABC transporters (Daumke and Knittler, 2001; Procko et al., 2006; Timachi et al., 2017; Tang et al., 2023). With its distinctive homologous NBD plus heterologous TMD architecture, YbhFSR provides a streamlined experimental example of this principle that eliminates confounding effects arising from intrinsic NBD asymmetry.

### 4.4 Functional implications of the fused NBD architecture

Unlike the Gram-positive prototypes BmrCD and TM287/288, which have separate NBD subunits, YbhF carries both NBDs on a single polypeptide chain. It is not obvious whether this fusion restricts the evolution of transporter asymmetry or reshapes how the NBDs communicate with the TMDs, but this question warrants explicit consideration.

Fused NBDs are actually quite common across bacterial ABC transporters. A useful comparison is the *E. coli* ribose transporter RbsABC: its RbsA subunit (501 residues) contains two tandem NBDs produced by an internal duplication (Bell et al., 1986). Barroga et al. (1996) showed that the first NBD acts as the principal catalytic site and that a single functional NBD is sufficient to maintain transport activity. Linton and Higgins (1998) argued, on the basis of phylogenetic analysis of *E. coli* ABC proteins, that heterodimeric TMD architectures can arise through gene duplication before fusion events — meaning functional asymmetry does not depend on whether the NBDs end up fused. YbhF fits this picture: NBD1 retains the canonical catalytic motifs, while NBD2 carries the degenerate LSGGM→LRGRA substitution, so asymmetry is clearly preserved even after fusion.

We do not yet have direct experimental data on how the fused NBDs couple to the TMDs in the YbhFSR complex. However, work on RbsABC suggests that a fused NBD pair containing one active and one degenerate site can still support productive coupling: ATP hydrolysis drives conformational changes in the substrate-binding protein (Erramilli et al., 2017). By extension, fusion in YbhF may well shift the coupling dynamics — for example, by making the dominant NBD the main driver of NBD–TMD communication — rather than breaking the process entirely.

That said, resolving how the fused NBDs actually coordinate with the TMDs in YbhFSR will require structural data. We have noted in the Limitations section that cryo-EM analysis of the full-length complex, combined with systematic site-directed mutagenesis, will be necessary to test these ideas properly.

## 5 Limitations

A major limitation of this study is that all functional interpretations regarding YbhS–YbhR asymmetry rely exclusively on cellular deletion phenotypes, including transcriptional profiling, antibiotic susceptibility testing, and efflux kinetic analysis. Consequently, potential physiological compensation or indirect secondary effects cannot be fully excluded, and direct biochemical and structural validation is presently lacking. Moreover, high-resolution structural data (cryo-EM or X-ray crystallography) for the YbhFSR complex are currently absent; thus, the proposed conformational differences between YbhS and YbhR are inferred from sequence analysis and cellular phenotypes instead of being directly visualized. Specifically, the substrate-binding properties of YbhR and the functional relevance of its candidate aromatic residues (Trp and Tyr) remain to be established through *in vitro* binding assays and site-directed mutagenesis. Structural analysis of the full-length YbhFSR complex would further help define the structural context of these residues. In addition, because complemented Δ*ybhS* and Δ*ybhR* strains could not be generated in this study, non-specific effects of the deletions cannot be formally excluded without complementation. We nevertheless note that Δ*ybhSR* and Δ*ybhFSR* showed phenotypes similar to Δ*ybhR*, which argues against an additional contribution of *ybhS*.

In addition, the regulatory mechanism driving uneven transcription across the *ybhFSR* operon is not yet defined. Whether this differential induction reflects variation in repressor affinity or intrinsic promoter architecture remains unclear. In addition, *gapA* was used as the reference gene for qRT-PCR without systematic validation under TC stress conditions. Although its Ct values remained stable across the tested TC concentrations, formal validation using established algorithms (e.g., geNorm, NormFinder) and evaluation of alternative reference genes were not performed, and this should be addressed in future work. Furthermore, evolutionary inference is restricted to Enterobacteriaceae; broader sampling across Proteobacterial lineages is needed to assess the universality of the observed TMD subfunctionalization pattern.

Future studies may aim to purify the full YbhFSR complex and determine its structure at atomic resolution by cryo-EM, allowing detailed comparison of YbhS and YbhR at the atomic level—particularly within substrate-binding pockets and NBD-TMD coupling interfaces. Such structural insights would complement cellular phenotypes and provide independent tests of the asymmetric functional model proposed here.

## 6 Conclusion

Transcriptional profiling, deletion phenotyping, and cross-species sequence comparisons reveal functional asymmetry between the TMD subunits of this Gram-negative ABC transporter, which couples fused NBDs to divergent TMD paralogs. Tetracycline stress triggers dose-dependent transcription of the *ybhFSR* operon and elicits robust compensatory activation upon inactivation of the major efflux system AcrAB–TolC. Functional assays indicate that YbhR is the major TMD subunit driving tetracycline efflux; deletion of YbhS has little or no effect on transport activity, supporting the notion of unequal functional contributions by the two paralogs. Evolutionary analyses further support this division. Across representative Enterobacteriaceae, YbhR exhibits higher interspecies conservation than YbhS in five of seven genera, consistent with stronger purifying selection on YbhR, although this difference did not reach statistical significance. The two subunits share only 24.9% full-length identity, consistent with extensive functional divergence.

YbhR therefore emerges as a candidate target for developing small-molecule inhibitors to counter YbhFSR-mediated tetracycline resistance. More broadly, YbhFSR offers a valuable model for dissecting how functional asymmetry is coordinated across NBD and TMD domains within a single ABC transporter complex.

## Data Availability

The original contributions presented in the study are included in the article/Supplementary material, further inquiries can be directed to the corresponding author.

## Supplementary material

SUPPLEMENTARY FIGURE S1 PCR amplification of the full-length coding sequences of *ybhR* and *ybhS*. Agarose gel electrophoresis (1%) of PCR products. Lane M, DNA molecular weight marker (DL2000); lane 1, *ybhS* amplicon (1,134 bp); lane 2, *ybhR* amplicon (1,107 bp). Both fragments were amplified from *E. coli* K-12 genomic DNA and subsequently confirmed by Sanger sequencing to be 100% identical to the NCBI reference sequences.
