# Supplementary material for: Functional asymmetry of the transmembrane subunits YbhS and YbhR in the tetracycline-inducible ABC transporter YbhFSR
Source: Front Microbiol. 2026 Oct 23;17:1959624. doi: 10.3389/fmicb.2026.1959624 (PMC13739795; doi:10.3389/fmicb.2026.1959624)
Supplement: Supplementary file 1 [file Data_Sheet_1.PDF]

Table S1. Primers used in this study

Primer name	Sequence (5'to3')	Size (bp)
ybhR-F	CGC[GGATCC]ATGTTTCATCGCTTATGGACGTT	32
ybhR-R	CCC[AAGCTT]TTACATCACCTTACGTCTAAACATCG	35
ybhS-F	CGC[GGATCC]ATGAGTAACCCGATCCTGTCCT	31
ybhS-R	CCC[AAGCTT]CTAATCCAGCCGACGTTTGG	29
K-*ybhR* F	CGACGTGGCTGAAAACCAAACGTCGGCTGGATTAGGGAGAA GAGCTTGAGCGATTGTGTAGGCTGGAG	68
K-*ybhR* R	CTTGCCGGCCCGGCGGTAGTGTCTTTGCTGGCGAAAGATAAG AAGTAACGGCTGACATGGGAATTAGC	68
K-*ybhS* F	AAGCCTTTATTCAGTTGATCCACGACTGGGATAAGGAGCATA GCATTGAGCGATTGTGTAGGCTGGAG	68
K-*ybhS* R	TCTTTGCGGATTAACGTCCATAAGCGATGAAACATGCTCTTCT CCTAACGGCTGACATGGGAATTAGC	68
T-*ybhR* F	GTTCAGCTGATTGAAGATGCGG	20
T-*ybhR* R	GATGTTGTTGGTCCACTGGTA	20
T-*ybhS* F	ATGGAGCAAGCCTTTATTCA	20
T-*ybhS* R	CGCCGTTATCTTCATCGTAG	20
Cm-F	TGAAACTCACCCAGGGATTG	22
Cm-R	ATAAATCCTGGTGTCCCTGT	22
Q-*ybhF*-F	ACCGGACATTCTGTTTCTCG	20
Q-*ybhF*-R	GACGCCTTTCTCTACCATGC	20
Q-*ybhS*-F	CGTACATTATTCCCGCTCGT	20
Q-*ybhS*-R	GATAAACATCACCGCCGAAG	20
Q-*ybhR*-F	AGCGCCTTTACTCATGTGCT	20

Primer name	Sequence (5'to3')	Size (bp)
Q-*ybhR*-R	GTATCCAGTTTGCGCGAGA	19
Q-*acrB*-F	CGATAACCTGATGTACATGTCC	22
Q-*acrB*-R	CCGACAACCATCAGGAAGCT	20
Q-*gapA*-F	CTGGTGCGAAGAAAGTGGTT	20
Q-*gapA*-R	TGATGCCGAAGTTATCGTTG	20

Primers were named according to the following conventions. Primers with the prefix "K-" were used to amplify targeting fragments for λ-Red homologous recombination; these fragments carry 45–50 bp homology arms flanking the target gene and FRT sites. Primers with the prefix "T-" were used for colony PCR to verify positive deletion mutants; these primers anneal to sequences within or flanking the target gene. Primers with the prefix "Q-" were used for quantitative real-time PCR (qRT-PCR). Primers with the prefix "Cm-" were used to detect the chloramphenicol resistance gene (cat).
